# Differences in the Clinical Characteristics of Early- and Late-Onset Asthma in Elderly Patients

**DOI:** 10.1155/2020/2940296

**Published:** 2020-01-27

**Authors:** Qin-Hua Liu, Xu Kan, Yong Bin Wang, Kai-xiong Liu, Dunhuang Zeng

**Affiliations:** ^1^Department of Respiratory Disease, Fujian Geriatric Hospital, Fuzhou 350003, China; ^2^Department of Geriatrics, Zhongshan Hospital, Fudan University, Shanghai 200032, China; ^3^Department of Respiratory Medicine, The Second Hospital of Shandong University, Jinan 250000, China; ^4^Department of Respiratory Disease, The First Affiliated Hospital, Fujian Medical University, Fuzhou 350005, China; ^5^Department of Respiratory and Critical Care Medicine, Ruijin Hospital, School of Medicine, Shanghai Jiaotong University, Shanghai, China

## Abstract

Differences between early-onset and late-onset asthma in elderly subjects have not been comprehensively described in China. We conducted a cross-sectional study to determine the phenotypic differences between early-onset asthma (EOA) and late-onset asthma (LOA) in elderly patients. We collected clinical and physiological data from 176 elderly patients with asthma. Participants were divided into two groups: EOA group and LOA group. Demographics, comorbidities, inflammatory parameters, lung function, severity, asthma control, and medication use among EOA and LOA elderly patients were compared. Elderly subjects with EOA had more atopic disease, a stronger positive family history of asthma, higher IgE, and exhaled nitric oxide levels as compared to those with LOA. In contrast, elderly subjects with LOA had lower lung function and more marked fixed airflow obstruction (FAO). Elderly subjects with LOA had a higher incidence of chronic obstructive pulmonary disease (COPD). No differences were observed in age, gender, BMI, history of smoking, severity, and asthma control between the two groups. Both similarities and differences exist between elderly subjects with EOA and those with LOA in China. Further work is required to determine the pathophysiological, clinical, and therapeutic implications for different asthma phenotypes in elderly subjects.

## 1. Introduction

Asthma is a major global health problem and poses a significant health and socioeconomic burden worldwide [1]. The prevalence of asthma differs geographically and varies ranging from 4% to 13% in elderly [2]. Asthma in the elderly is associated with higher morbidity and mortality than asthma in younger subjects [3]. Given the rising prevalence of asthma and increasing life expectancy, an elaborate and efficient approach to asthma diagnosis and management tailored to well-defined phenotypes is required. Asthma affects a large number of elderly patients in China. Many challenges exist in the recognition, diagnosis, and treatment of asthma in this population [4].

Asthma is a phenotypically heterogeneous disease characterized by diverse clinical characteristics and inflammatory pathologies [5]. There are distinct phenotypes of asthma in elderly patients; however, unlike in younger patients, they are not well characterized [6]. Some studies comparing EOA and LOA in this population have been conducted in Europe and America [7, 8], but comprehensive evaluation of clinical characteristics and outcomes in the elderly in China is still lacking. In the current study, we aim to determine whether phenotypic differences exist between EOA and LOA in elderly subjects so as to improve our understanding of the clinical characteristics and pathogenesis and facilitate improvement of targeted therapies.

## 2. Methods

### 2.1. Study Design

This was a cross-sectional study determining whether phenotypic differences exist between EOA and LOA in elderly patients. Elderly subjects ≥65 years with asthma were consecutively recruited from the outpatient clinics of Fujian Geriatric Hospital, the First Affiliated Hospital of Fujian Medical University, the Second Affiliated Hospital, Shandong University, and Zhongshan Hospital Affiliated Fudan University. Study enrollment took place from January 2017 to December 2017. All participants had stable disease with unchanged asthma medication for at least 4 weeks before enrollment. The diagnosis was based on typical symptoms and was confirmed by objective lung function measurements according to the criteria proposed by the GINA. The diagnosis was made by respiratory physicians from tertiary hospitals in Fujian province. The subjects were excluded if they had cognitive impairment or an inability to attend study visits. All participants underwent serum IgE test, PFT, and FeNO test. The local research ethics committee approved the current study, and written informed consent was obtained from all participants.

### 2.2. Data Collection and Assessments

Clinical data were collected and included basic variables (sex, date of birth, age of onset, family history of asthma, smoking history, BMI, and residential area), atopy, comorbidities, symptoms, asthma control, therapeutic interventions, PFT, and inflammatory parameters (serum IgE and FeNO). PFT was performed using a lung function machine (JAEGER; German) by two experienced technicians in accordance with the CTS Guidelines of Pulmonary Function Test [9]. Atopy was assessed by SPT with allergen extracts. Comorbidities were based on participants' self-reporting and available medical records. Depression was assessed using the Hospital Anxiety and Depression Scale (HADS) [10]. FeNO level was measured using the NO electrochemical equipment (NIOX VERO; Aerocrine AB, Solna, Sweden) by two experienced technicians.

### 2.3. Definitions

EOA was defined as asthma diagnosed prior to 45 years of age, whereas LOA was defined as asthma diagnosed at the age of 45 years or older. CVD events were defined as the occurrence of acute coronary syndrome, stable angina, stroke, coronary revascularization, heart failure, or CVD death. FAO was defined as an FEV1/FVC ratio <0.7 on all of three pulmonary function tests despite the use of high-dose ICS or LABA [11]. Percentage predicted values (% pred) were calculated based on the reference values for healthy Chinese adults. The dose range of high-dose ICS for adults was consistent with GINA guidelines. Asthma severity was classified as mild, moderate, or severe according to the ATS definition. Asthma control status was assessed using the ACT and classified as well-controlled (≥20), partially controlled (16 to <20), and poorly controlled asthma (5 to <15) [12].

### 2.4. Statistical Analysis

We performed statistical analysis using SPSS software, Version 21.0 (SPSS Inc., Chicago, IL, USA). Results were expressed as mean ± standard deviation (SD) for quantitative variables, or median (interquartile range) for nonnormal variables where appropriate, or number (%) of patients. Comparative analysis of variance between EOA and LOA groups was performed using Student's *t*-test. A test of proportions between groups was performed using the Pearson chi-squared test. Statistical significance was considered to be present for *p* values <0.05.

## 3. Results

A total of 176 participants aged ≥65 years were included. Ninety-two (52.3%) and eighty-four (47.7%) had early-onset and late-onset asthma, respectively. Demographic and clinical features were listed in Table 1. All participants were Han Chinese. The mean age of participants was 70.98 years. Patients were mostly females (94/176, 53.41%). The percentages of never, previous, and current smokers were 59.1%, 23.3%, and 19.3%, respectively. No differences were observed in age, gender, BMI, history of smoking, or living region between the groups.

Atopy was observed in 23.9% of elderly asthmatic patients. Seventy-eight patients (78/176, 44.4%) had an FH of asthma. EOA was more strongly associated with atopy and FH of asthma (*p*=0.032; *p*=0.020). A higher prevalence of allergic rhinitis and eczema was noted in the EOA group (*p*=0.012; *p*=0.050). Serum IgE was also higher in subjects with EOA (151.27 ± 99.22 vs 82.43 ± 41.64, *p*=0.001). The average FeNO value in elderly patients with asthma was 38.64 (17.98) ppb. The FeNO value was higher in EOA group compared with LOA group (45.58 ± 21.85 vs 31.05 ± 6.83, *p*=0.001). The prevalence of COPD in our cohort was 21.6%. In the current study, 53.4% (94/176) of patients had CVD and 7.4% (13/176) had depression. COPD was more common in elderly patients in the LOA group (*p*=0.012). No significant difference was found when comparing the percentage of CVD, depression, and bronchiectasis, and anxiety between the two groups (*p*=0.656, *p*=0.906, *p*=0.760, and *p*=0.227, respectively).

There were no significant differences in asthma control and severity of disease between the EOA and LOA groups. Based on ACQ cut-points, 26.7% (47/176) of patients had uncontrolled asthma, and 38.6% and 34.7% of patients achieved partial or complete asthma control, respectively. Over the preceding 12-month period, 20.5% (36/176), 23.3% (41/176), and 25.6% (45/176) of patients were admitted to hospitals, visited the ED, or had unscheduled doctor visit, respectively. Fifteen patients had a history of intubation. A greater proportion of patients received high dose of ICS/LABA in the LOA group (*p*=0.025). No significant difference in asthma attack between EOA and LOA groups was noted.

Spirometry values of FEV1 % predicted (*p*=0.000), FVC % predicted (*p*=0.006), and FEV1/FVC ratio (*p*=0.005) were found to be significantly lower in the LOA group as compared to the EOA group (Table 2). Patients from the LOA group had a significantly lower diffusing capacity for carbon monoxide (DLCO % predicted) as compared to those from the EOA group (*p*=0.006). Data of technically acceptable post-BD spirometry were obtained from 164 (93.2%) participants. Of these, 37 (22.56%) had FAO. FAO in the LOA group was higher compared with the EOA group (*p*=0.017).

## 4. Discussion

In this study, we firstly described the differences in the demographic, clinical, and pathophysiological characteristics between EOA and LOA in an elderly Chinese population in mainland China. Asthma in the elderly consists of multiple and distinct phenotypes, subgroups of patients with special characteristics, which are poorly understood since only a small minority of studies have focused on such population. In contrast to EOA, less was known about the characteristics of LOA. Approximately half of the patients included in the study were EOA persisting into adulthood, and half were LOA. Our study reconfirms some previous observations in elderly subjects with asthma but adds particular information from southern China. Age of asthma onset is a crucial factor in distinguishing different adult asthma phenotypes. The cutoff age for the diagnosis of EOA or LOA was not consistent [5, 13–15]. We took 45 years as a cutoff based on a prior longitudinal study [13].

In line with previous studies, females accounted for the majority of elderly subjects with asthma. However, no clear gender predominance between EOA and LOA was noted in our study. Female sex hormones and gender-specific differences in allergic sensitivities have been shown to exist in elderly subjects with asthma [16, 17]. The active smokers in our study accounted for a small minority of the sample. Long-term illness and recurrent asthma attacks in EOA group might encourage patients to avoid cigarette smoke. However, in our study, a number of subjects with FAO in the LOA group had a history of cigarette smoking. The role of smoking in LOA should be given special attention due to an increased risk of poor asthma control and corticosteroid insensitivity [18, 19]. Smokers can have FAO or an exacerbation-prone phenotype. Although smoking in both groups was not frequent, it is difficult to exclude the fact that the individual could have been affected by second-ever third-hand smoking on account of the large number of smokers in China [20].

We have also found age-related decrease in total IgE and fewer positive SPT responses and less concomitant AR or AD. These findings are in agreement with other studies [21]. Atopic asthma represents the most common form of asthma and is characterized by eosinophilic airway inflammation associated with specific IgE antibodies sensitization to various allergens. The role of environmental exposures and allergy in LOA is largely unknown. Atopy that was inversely correlated with age of asthma onset may not be mechanism of disease in elderly patients with LOA [22]. Aging is associated with modifications of the immune system, namely, immunosenescence. This could contribute to the decline of allergen sensitization in elderly populations [23]. AR was associated with asthma in general populations, supporting the “united airway” hypothesis. However, data on prevalence of AR for the elderly population are scarce [24]. The overall prevalence of AR in the current study was 43.18%, which outclassed the 17.7% prevalence of AR in the Chinese adult population [25]. Genetic predisposition has a considerable role in the onset of asthma. GWAS has demonstrated the associations between specific genetic variants and susceptibilities to asthma onset in childhood [26]. The role of genetic predisposition in LOA is less definite than that in EOA. EOA and LOA might be affected by the interaction between genetic variations and environmental microbes [27]. While family history of asthma increased overall risk of asthma, the strong effect of inheritance pattern was more obvious in subjects with EOA [28, 29].

Comorbidities might exert a negative impact on asthma phenotypes, severity, and asthma attack. Whether comorbidities are causally related or just occasional conditions or trigger factors remained unclear. Asthma was associated with an increased risk of CVD and all-cause mortality [30]. Chronic airway inflammation might elicit systemic inflammation and increase vulnerability to CVD. LOA, but not EOA, was associated with an increased risk of CVD events [31]. Elderly patients with coexistence of asthma and COPD experienced frequent exacerbations and had a poor quality of life, a more serious deterioration in lung function, and higher mortality than asthma alone. Prevalence of coexisting COPD in asthma in our study was lower than that in other studies [32, 33]. The coexistence of bronchiectasis and asthma has also been observed in many patients, especially in Chinese asthma populations [34, 35]. The exact causal relationship between bronchiectasis and asthma has not been fully understood. Presumably mucus hypersecretion, recurrent infection, bacterial colonisation, oxidative stress, and airway remodeling are possible mechanisms for the coexistence of asthma and bronchiectasis [36]. Obesity affects a minor proportion of subjects in our study. Obese elderly individuals were more likely to suffer from asthma [37]. Age of asthma onset modified the association between obesity and asthma [38]. Depression contributed to poor outcomes in older asthmatics and may represent a sequela of chronic disease [39]. Asthma may impose a significant burden on emotional wellbeing, mainly because of the fear of shortness of breath. Depression might alter airway efferent neural activity, which leads to airway constriction [40]. Nearly one-third of our participants had an anxiety disorder, which is quite striking and similar to recent studies [41].

Previous studies concerning the lung function difference between early- and late-onset phenotypes have provided conflicting results [14]. Our study showed that adults with LOA had higher proportion of FAO, which was in line with other studies [42, 43]. Physiological changes within organs, tissues, and cells in elderly subjects resulted in diminished lung functional reserve. Patients with LOA have been found to have higher lung eosinophils compared to those with EOA and may have a more progressive loss in lung function [44]. The decrease in DLCO % predicted might be associated with physiological aging, emphysema, and asthmatic airway remodeling [32]. Our findings identified the prevalence of FAO in older adults with asthma at a fairly high rate of 30%, which is similar to the study by Bennett GH [45]. Active smoking, airway remodeling, bronchiectasis, postinfectious linear fibrosis, and air-trapping contribute substantially to FAO that presented a distinct elderly asthmatic phenotype. FAO was associated with frequent exacerbations and worsened asthma control [45, 46]. FeNO that indicates ongoing eosinophilic airway inflammation has been extensively studied in adults and children with asthma, but little is known about the role of FeNO in elderly asthmatics [47]. The phenotype of EOA in our cohort may be largely associated with eosinophilic inflammation.

Asthma seems to be more difficult to control in the elderly. The proportion of patients with uncontrolled asthma was substantially greater in China [48]. Elderly subjects with EOA and LOA have similar overall disease severity, ED attendance, and hospital admissions. Some primary care physicians, especially in rural and remote regions in China, often prescribe oral steroids for elderly patients with asthma to control the symptoms. Given the low cost of theophylline, it is widely used for relieving shortness of breath in Chinese patients especially in those with LOA [49]. Our study found that triple controller therapy and high-dose ICS were more frequent in LOA, which might indicate that LOA exhibits a worse treatment response than EOA [50]. In our study, hospitalization and ED attendance rates in our cohort were much higher than those reported in other studies from United States, which were similar to those seen in other asthma cohorts from China [51]. Given unsatisfactory asthma control in our region, asthma education focused on patients and primary care physicians should be further encouraged.

Substantial heterogeneity in hereditary pattern exists between EOA and LOA. Both hereditary and environmental factors contribute to the occurrence of adult asthma. EOA or LOA may not be a single entity. There may be different endotypes within them. Although sparse data exist on the phenotypic profiles of asthma in elderly patients, limited data showed that LOA in elderly is more heterogeneous when compared with EOA.

Several limitations of this study deserve to be acknowledged. First, it is an observational cross-sectional analysis, and causal associations for the collected data cannot be confirmed. Our cohort is not too big to explore the character of special clusters such as severe asthma. Second, the possibility of recall bias should be noted. Age of onset of asthma, recent health care utilization, and comorbidities were possibly subject to recall bias. Third, geographical difference and socioeconomic status should also be taken into consideration before one extrapolates these findings to the whole nationwide. In addition, these patients referred to a tertiary center perhaps represented a more highly selected or severe patients. Fourth, we did not perform sputum cytology and computed tomography to explore airway inflammation and airway remolding. Whether the increased presence of airway neutrophils contributes to greater asthma severity or LOA in the elderly remained unknown. Whether the unique structural alterations are observed between EOA and LOA in elderly subjects also remained unclear.

## 5. Conclusion

Our study reaffirms that age at onset is a key determinant of different phenotypes in the elderly Chinese adults with asthma. Elderly subjects with LOA have different clinical and physiological characteristics compared to those with EOA. Considerable mechanistic heterogeneity exists in such a complex population with multiple and diverse phenotypes. There is a great need for further clinical, basic, and translation research concerning the phenotypic and mechanistic subtypes of elderly asthmatic to achieve personalised management.

## Figures and Tables

**Table 1:** Difference in demographics and comorbidities between EOA and LOA in elderly subjects^*∗*^.

Variables	Total (*n* = 176)	EOA (*n* = 92)	LOA (*n* = 84)	*p* value
Age	70.98 ± 6.49	70.3 ± 77.10	71.65 ± 5.702	0.001
Female	94 (53.4%)	51	43	0.573
Rural region	64 (36.4%)	31	33	0.441
Smoking status				
Never	104 (59.1%)	60	44	0.084
Former	41 (23.3%)	20	21	0.609
Current	31 (19.3%)	12	19	0.096
BMI, kg/m^2^	22.57 ± 2.43	22.31 ± 2.27	22.85 ± 2.58	0.140
FH asthma	78 (44.4%)	48	30	0.028
Atopy	42 (23.9%)	28	14	0.032
Allergic rhinitis	76 (43.8%)	48	28	0.012
Allergic eczema	50 (26.9%)	32	18	0.050
COPD	38/29 (21.6%)	13/10	25/19	0.012
Bronchiectasis	36 (20.5%)	18	18	0.760
CVD	51 (29.0%)	28	23	0.656
Depression	13 (7.4%)	7	6	0.906
Anxiety	56 (31.8%)	33	23	0.227
IgE	118.41 ± 84.44	151.27 ± 99.22	82.43 ± 41.64	0.001
FeNO, mean (SD) ppb	38.64 ± 17.98	45.58 ± 21.85	31.05 ± 6.83	0.001
Controlled	61 (34.7%)	30	31	0.550
Uncontrolled	47 (26.7%)	26	21	0.625
Partly controlled	68 (38.6%)	36	32	0.888
Asthma severity				
Mild	58 (33.0%)	30	28	0.919
Moderate	78 (44.3%)	44	41	0.252
Severe	30 (17.0%)	18	15	0.784
Medications				
Number on ICS/LABA	153 (86.9%)	81	72	0.647
Number on high-dose ICS/LABA	32 (18.2%)	11	21	0.025
Number on LAMA				
Maintenance oral prednisolone	7 (4.0%)	4	3	0.792
Maintenance oral theophylline	47 (26.7%)	20	27	0.119
Unscheduled doctor visit in past one year	45 (25.6%)	25	20	0.609
ED (<24 h) in past one year	41 (23.3%)	23	18	0.576
Hospitalizations (>24 h) in past one year	36 (20.5%)	19	17	0.946
History of intubation	15 (8.5%)	8	7	0.931

^*∗*^All parametric data are expressed as mean (standard deviation) unless stated otherwise.

**Table 2:** Difference in lung function between EOA and LOA in elderly subjects^*∗*^.

	Total (*n* = 176)	EOA (*n* = 92)	LOA (*n* = 84)	*p* value
	Mean ± SD	Mean ± SD	Mean ± SD	
FEV1 (% predicted)	0.79 ± 0.20	0.84 ± 0.18	0.73 ± 0.20	0.001
FVC (% predicted)	0.82 ± 0.15	0.85 ± 0.16	0.79 ± 0.13	0.006
FEV1/FVC	0.72 ± 0.18	0.75 ± 0.16	0.69 ± 0.20	0.005
FAO (no, %)	37	13	24	0.017
DLCO (% predicted)	0.87 ± 0.11	0.90 ± 0.12	0.83 ± 0.08	0.006

^*∗*^All parametric data are expressed as mean (standard deviation) unless stated otherwise.

## Data Availability

The data used to support the findings of this study are mostly included within the article. The data of included patients are available from the corresponding author upon request.

## Abbreviations

ACT: Asthma control test
AD: Atopic dermatitis
AR: Allergic rhinitis
ATS: American Thoracic Society
BMI: Body mass index
COPD: Chronic obstructive pulmonary disease
EOA: Early-onset asthma
FAO: Fixed airflow obstruction
F ENO: Fractional exhaled nitric oxide
FEV1: Forced expiratory volume in 1 second
FH: Family history
FVC: Forced vital capacity
GINA: Global Initiative for Asthma
GWAS: Genome-wide association study
ICS: Inhaled corticosteroid
IgE: Immunoglobulin E
LABA: Long-acting *β*_2_-receptor agonist
LOA: Late-onset asthma
MD: Mean difference
PFT: Pulmonary function test
SPT: Skin prick testing.
